# Expression of Selected *Ginkgo biloba* Heat Shock Protein Genes After Cold Treatment Could Be Induced by Other Abiotic Stress

**DOI:** 10.3390/ijms13055768

**Published:** 2012-05-15

**Authors:** Fuliang Cao, Hua Cheng, Shuiyuan Cheng, Linling Li, Feng Xu, Wanwen Yu, Honghui Yuan

**Affiliations:** 1 Economic Forest Germplasm Improvement and Comprehensive Utilization of Resources of Hubei Key Laboratory, Huanggang Normal University, Huanggang 438000, China; E-Mails: chenghua1437@sina.com (H.C.); lilinling1437@126.com (L.L.); xufeng198@126.com (F.X.); 2 College of Forest Resources and Environment, Nanjing Forestry University, Nanjing 210037, China; E-Mail: youeryuww@163.com; 3 College of Chemistry and life science, Huanggang Normal University, Huanggang 438000, China; E-Mail: yuan20110826@126.com

**Keywords:** heat shock proteins, SSH, cold stress, heat stress, abiotic stress, *Ginkgo biloba*

## Abstract

Heat shock proteins (HSPs) play various stress-protective roles in plants. In this study, three *HSP* genes were isolated from a suppression subtractive hybridization (SSH) cDNA library of *Ginkgo biloba* leaves treated with cold stress. Based on the molecular weight, the three genes were designated *GbHSP16.8*, *GbHSP17* and *GbHSP70*. The full length of the three genes were predicted to encode three polypeptide chains containing 149 amino acids (Aa), 152 Aa, and 657 Aa, and their corresponding molecular weights were predicted as follows: 16.67 kDa, 17.39 kDa, and 71.81 kDa respectively. The three genes exhibited distinctive expression patterns in different organs or development stages. *GbHSP16.8* and *GbHSP70* showed high expression levels in leaves and a low level in gynoecia, *GbHSP17* showed a higher transcription in stamens and lower level in fruit. This result indicates that *GbHSP16.8* and *GbHSP70* may play important roles in *Ginkgo* leaf development and photosynthesis, and *GbHSP17* may play a positive role in pollen maturation. All three *GbHSPs* were up-regulated under cold stress, whereas extreme heat stress only caused up-regulation of *GbHSP70*, UV-B treatment resulted in up-regulation of *GbHSP16.8* and *GbHSP17*, wounding treatment resulted in up-regulation of *GbHSP16.8* and *GbHSP70*, and abscisic acid (ABA) treatment caused up-regulation of *GbHSP70* primarily.

## 1. Introduction

Heat shock protein (HSP) is a type of specific stress protein produced by living organisms in response to high temperatures and other environmental stresses. Abundant HSP expression could remarkably improve the survivability and endurance of cells to environmental stress or damage [1]. HSPs can be classified into five families based on molecular weight, namely, HSP100, HSP90, HSP70, HSP60, and small molecule sHSP [2].

Previous studies had shown that the accumulation of HSPs plays a pivotal role in abiotic stress responses in plants [3–5]. Most HSPs function as molecular chaperones in maintaining homeostasis of protein folding and are thought to be responsible for the acquisition of thermo tolerance [6]. Aside from high temperature, low temperature [7], drought stress [8], heavy metal ions [9], high salinity [10], anaerobic environments [11], diseases and pests [12], ultraviolet light [13], superoxide ions [14,15], mechanical injury, SA [16,17] and abscisic acid (ABA) treatment [5] can all induce HSP generation. Moreover, HSPs were shown to be involved in many steps of cell apoptosis [18,19]; the protective effects of the chaperone machinery, in which different HSPs or chaperones acted cooperatively [2]. In the absence of environmental stresses, the expressions of some HSPs were shown to be developmentally [20] or tissue-specifically regulated [5]. Transgenic plants overexpressing *HSP* genes exhibited improved tolerance to heat, cold stress and drought stress [21–23].

Currently, *Ginkgo biloba* is one of the most popular functional plants, particularly as a medicinal plant. Extracts of *G. biloba* leaves contained active compounds such as flavonoids and terpene lactones (ginkgolides and bilobalide), which may induce an increase in peripheral and cerebral blood flow [24,25]. *Ginkgo* has important economic and medicinal values, and its plantation scope is expanding gradually. The extreme temperature in *G. biloba*’s natural habitat does not exceed 40 °C or fall below 4 °C, which affects its regional expansion. *G. biloba* has survived all kinds of complex climatic environments for millions of years; it has shown strong adaptability and has changed little in morphology. The survival of *G. biloba* is more or less related to its strong tolerance to environmental stresses [26]. In this study we isolated three novel *HSP* genes after cold treatment of *Ginkgo* leaves. Expression analysis indicated that the selected *Ginkgo biloba* heat shock protein genes from low temperature seedling could be induced by heat stress and other abiotic stress. A study of the cloning and expression of the heat shock protein genes in *G. biloba* will help reveal the coping mechanism of this plant to the environment and the physiological mechanism of resistance. In addition, it can provide the theoretical foundation and gene resources for cultivating resilient forests using gene engineering.

## 2. Results and Discussion

### 2.1. Results

#### 2.1.1. Cloning and Sequence Analyses of Three Genes Encoding Heat Shock Protein from *Ginkgo biloba*

**Figure 1:**
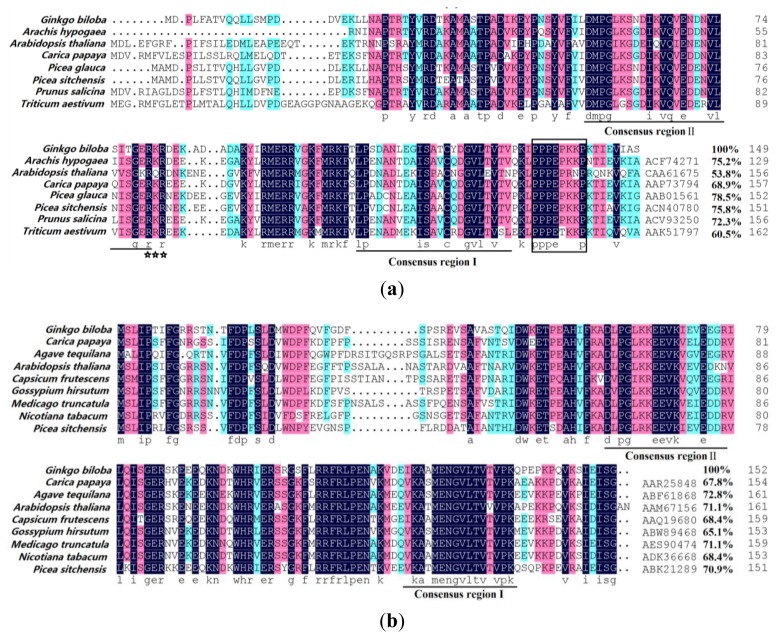

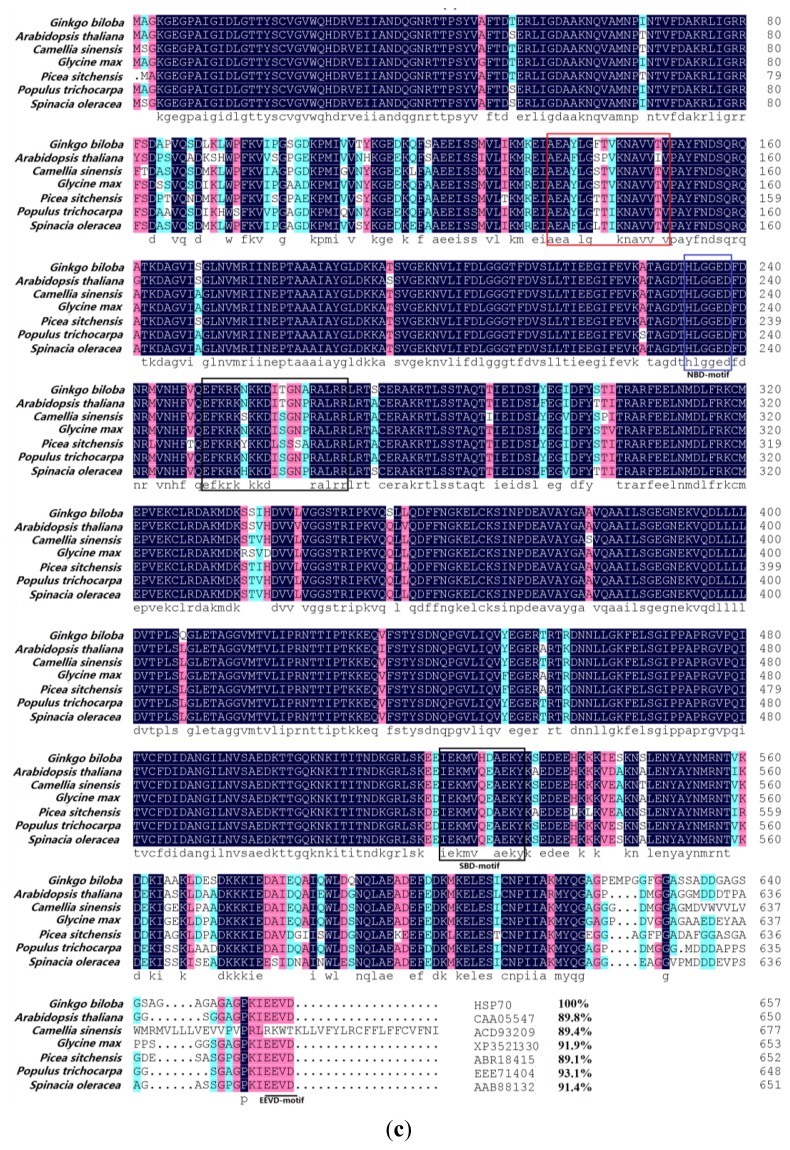
Protein sequence alignment of *Ginkgo* heat shock protein (HSP) with other HSPs. Two consensus regions are underlined and a putative nuclear localization signal is indicated by asterisks. A polyproline motif at the carboxyl end of proteins is boxed. Following the aligned sequences is the accession numbers and homology percentage. (**a**) HSP 16.8; (**b**) HSP 17, (**c**) HSP 70.

To investigate the molecular events of *Ginkgo* development and environments, two cDNA libraries were constructed using mRNAs isolated from *Ginkgo* leaves after cold shock treatment or in normal growing conditions. Three ESTs (expressed sequence tag) were isolated from the cold-treated cDNA library and the full-length cDNA was obtained by RACE. Sequencing revealed that the full length of the three *HSP* genes were 905 bp, 623 bp, and 2408 bp (Figure S1), respectively. According to their approximate molecular weight, the three *GbHSPs* studied in this work were named *GbHSP16.8*, *GbHSP17*, *GbHSP70*. The ORFs of the full length *GbHSP16.8*, *GbHSP17*, and *GbHSP70* were 450, 459, and 1974 bp, respectively. Three polypeptide chains containing 149 amino acids (Aa), 152 Aa, and 657 Aa were respectively encoded and their corresponding molecular weights were predicted as follows: 16.67 kDa, 17.39 kDa, and 71.81 kDa. The pIs were predicted as: 6.55, 5.83, 5.12, respectively. The three HSPs exhibited a high similarity with the amino acids of other HSPs (Figure 1). The comparison of BLASTP results in NCBI showed that *GbHSP70* had more than 93.1% similarity with HSP70 of *Populus trichocarpa* (EEE71404), 91.9% similarity with *Glycine max* (XP3521330) and 91.4% similarity with *Spinacia oleracea* (AAB88132); The amino acid sequence of the GbHSP16.8 shared 78.5% identity with *Picea glauca* (AAB01561)*,* 75.8% with *Picea sitchensis* (ACN40780), 75.2% with *Arachis hypogaea* (ACF74271), 72.3% with *Prunus salicina* (ACV93250), 68.9% with *Carica papaya* (AAP73794), 60.5% with *Triticum aestivum* (AAK51797), 53.8% with *Arabidopsis thaliana* (CAA61675); The GbHSP17 shared 72.8% homology with *Agave tequilana* (ABF61868), 71.1% with *Arabidopsis thaliana* (AAM67156) and *Medicago truncatula* (AES90474), 70.9% with *Picea sitchensis* (ABK21289), 68.4% with *Capsicum frutescens* (AAQ19680) and *Nicotiana tabacum* (ADK36668), 67.8% with *Carica papaya* (AAR25848), 65.1% with *Gossypium hirsutum* (ABW89468).

**Figure 2:**
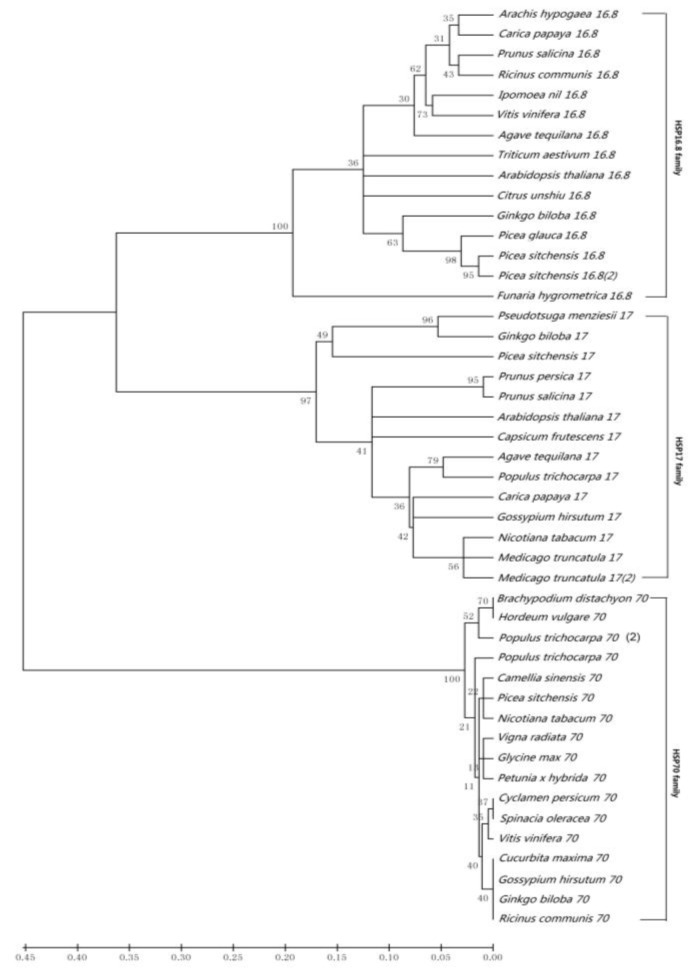
Gene tree of the three *Ginkgo biloba* HSPs. Gene trees were constructed using the software Molecular Evolutionary Genetics Analysis (MEGA) Version 4.0 by the neighbor-joining method with pairwise deletion and the poisson correction model. Bootstrap support values for each node are shown (percentage of bootstrap trees supporting the node, out of 1000 trees). Accession numbers for all sequences are as listed here. **Hsp16.8 family:** *Arabidopsis thaliana* CAA61675, *Arachis hypogaea* ACF74271, *Carica papaya* AAP73794, *Citrus unshiu* BAK61844, *Picea glauca* AAB01561, *Picea sitchensis* ACN40780, *Picea sitchensis(2)* ABK26390, *Prunus salicina* ACV93250, *Triticum aestivum* AAK51797, *Ipomoea nil* AAB39335, *Vitis vinifera* XP 2280485, *Agave tequilana* ABF61870, *Funaria hygrometrica* CAC81966, *Ricinus communis*, XP 2516106; **HSP17 family:** *Agave tequilana* ABF61868, *Arabidopsis thaliana* AAM67156, *Capsicum frutescens* AAQ19680, *Carica papaya* AAR25848, *Gossypium hirsutum* ABW89468, *Medicago truncatula* AES90474, *Medicago truncatula(2)* AES75921, *Nicotiana tabacum* ADK36668, *Picea sitchensis* ABK21289, *Populus trichocarpa* EEE89499, *Prunus persica* AAR99375, *Prunus salicina*, ACV93249, *Pseudotsuga menziesii* CAA63570; **HSP70 family:** *Brachypodium distachyon* XP 3558228, *Hordeum vulgare* BAJ86014, *Populus trichocarpa* EEE71403, *Populus trichocarpa* EEE71404, *Camellia sinensis* ACD93209, *Picea sitchensis* ABR18415, *Nicotiana tabacum* AAR17080, *Vigna radiata* AAS57912, *Glycine max* XP 3521330, *Petunia hybrida* CAA30018, *Cyclamen persicum* ABP35942, *Spinacia oleracea* AAB88132, *Vitis vinifera* CAN81694, *Cucurbita maxima* AAN86274, *Gossypium hirsutum* ACJ11741, *Ricinus communis* EEF34649.

An alignment of the deduced protein sequence of GbHSP16.8 and GbHSP17 with other plant cytosolic class I and class II sHSPs are shown in Figure 1a,b. Comparison with other plant sHSPs representing the two cytoplasmic subfamilies revealed that *GbHSP17* cDNAs coded for cytoplasmic class I sHSP subfamily members, *GbHSP16.8* for class II. As shown in Figure 2, within each class there was more similarity to proteins of other organisms belonging to the same class than to proteins of the other class. The sequence of the *N*-terminal domain of plant cytosolic class II sHSP was divergent, which may partially account for their functional multiplicity among different plant species. By contrast, all plant cytosolic *sHSP* share a conserved *C*-terminal domain of about 90 Aa called ACD or heat shock domain, which can be further divided into two subdomains, Consensus I Pro-A(14)-Gly-Val-Leu and Consensus II Pro-A(14)-Val/Leu/Ile-Val/Leu/Ile of the carboxyl terminal. A putative nuclear localization signal (RKR) and a polyproline motif (PPPEPKKP) are only found at the *C*-terminal of *GbHSP16.8*.

The secondary structure of HSP70 has α-helical segments, immediately followed by a β-sandwich subdomain. The *C*-terminal 10-kDa subdomain is α-helical while the other subdomains are β-sheets. Two regions are mutually dependent. The structure of the β-sandwich is consistent with the β-bridge at the *N*-end. A detailed analysis of the ATPase domain (nucleotide binding domain, NBD) and polypeptide-binding (substrate binding domain, SBD) [27] demonstrated that the side chains of amino acids critical for protein functions have conserved positions (Figure 2c). In particular, within the *N*-terminal NBD, the HLGGED motif in positions 234–239, critical for ATP/ADP binding, is conserved [28]. The same holds true for residues V415, M416, L419, I420, A496 and D498 and the motif IEKMVHDAEKY (521–531) within the *C*-terminal SBD, critical for peptide binding [29]. Furthermore, within the *N*-terminal, the EEVD motif in positions 654–657 is the signature cytosolic HSP70-specific motif [30].

**Figure 3:**
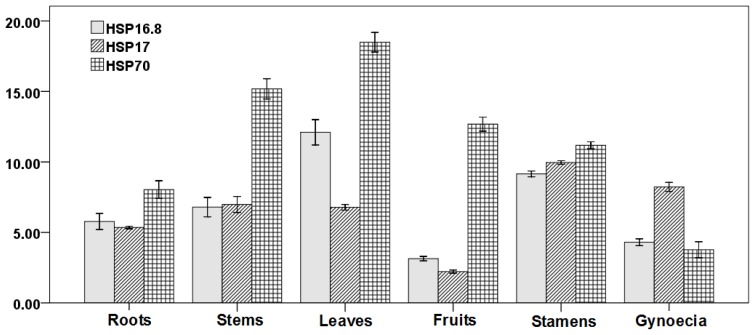
Analysis of QRT-PCR for tissue-specific transcription of *GbHSP* accumulation after 2 h heat shock.

To classify the three GbHSPs, we generated a phylogenetic tree using Molecular Evolutionary Genetics Analysis (MEGA) Version 4.0 by the neighbor-joining method (Figure 2). Phylogenetic analysis and amino acid sequence alignment indicated that the Aa sequence of GbHSP16.8 and GbHSP17 has high homology with the cytoplasmic II sHSPs of other plants. As shown in Figure 3, based on the amino acid sequence homology, the three *GbHSPs* genes were divided into three classes. *GbHSP70* belonged to HSP70 family while GbHSP16.8 and GbHSP17 belonged to two cytoplasmic sHSP subfamilies.

#### 2.1.2. Expression Analysis of Three *GbHSPs* Genes in Different Tissues

The expression of the three *GbHSPs* genes was analyzed using QRT-PCR. The three *GbHSPs* genes exhibited diverse expression in different organs, although expression could be detected in all organs we checked (Figure 3). Gb*HSP16.8* was highly expressed in *Ginkgo* leaves, stamens, stalk, root, and pistil, and was less abundant in the fruit section. *GbHSP17* was predominantly expressed in stamens, pistil, stalk, leaves, and roots, and moderately expressed in the fruit. *GbHSP70* had the highest expression level among the three *HSP* genes with the highest expression level in leaves, followed by stalks, fruits, stamens, and roots. The lowest expression level of *GbHSP70* was observed in pistil. In the current study, *GbHSP16.8* and *GbHSP17* were predominantly expressed in stamens, and would play roles in pollen development and pollen maturation. Usually reproductive organs are much more sensitive to heat stress than other organs [5]. *GbHSP70* was more highly expressed in leaves and stems than other organs, indicating that these genes may play some roles in maintaining the normal leaf functions, such as respiration and photosynthesis.

#### 2.1.3. Expression Profiles of the Three *HSPs* under Low Temperature or Heat Shock Treatments at Seedling Stage

**Figure 4:**
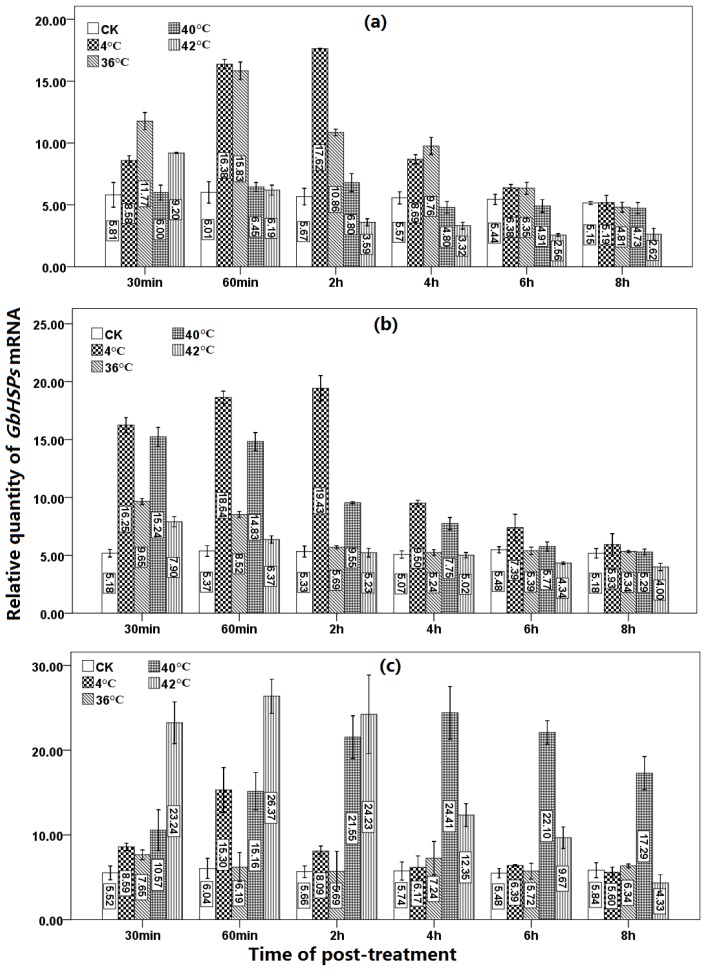
Under different stress temperatures, mRNA expression levels of the three *GbHSPs* were analyzed by real-time quantitative RT-PCR, *G. biloba GAPDH* gene was used as the internal control. Relative expression levels are shown for: (**a**) The relative expression levels of *GbHSP16.8* at different times after stress induction. (**b**) The relative expression levels of *GbHSP17* at different times after stress induction. (**c**) *GbHSP70* genes expression levels.

The changes in expressions levels of the three *GbHSP* genes from *G. biloba* were determined in response to low and high temperatures (Figure 4, Table S1). Specific primers for the three *GbHSPs* were selected and used in QRT-PCR to measure the dynamic changes in expression. The expressions of *HSP16.8* and *HSP17* were induced by low temperature (4 °C); whereas, the transcription level of HSP70 was only slightly affected. The transcription level of *HSP16.8* increased sharply to approximately 1.48 times higher than the control level (CK on Figure 4) after 30 min at low temperature, and to approximately 2.73 times after 60 min. The highest level, 3.11 times that of the control, was achieved at 2 h. After 8 h, the transcription level of *GbHSP16.8* decreased rapidly until it reached the control level. For *GbHSP17* at 4 °C, the transcript level rose rapidly to approximately three times that of the control after 30 min, reaching a peak of 3.65 times the control level at 2 h. After 8 h, the transcription level of *GbHSP17* decreased sharply to 1.15 times that of the control. The response of *GbHSP70* to low temperature was much slower than the other two HSPs. After 30 min at low temperature, the transcription level rose to 1.56 times that of the control and then peaked after 60 min to 2.53 times that of the control. The transcription level then decreased rapidly to that of the control level.

High temperatures (36, 40 and 42 °C) had different effects on the expression of the three *HSPs* in *G. biloba*. At 36 °C, the transcription level of *GbHSP16.8* rose to twice that of the control after 30 min and reached a peak after 60 min (Figure 4a). The transcription level decreased slowly to a level slightly lower than that of the control at 8 h. At 40 °C, the transcription level of *GbHSP16.8* showed no significant difference after 30 min; however, at 2 h, a slight increase was observed, after which the transcription level decreased slowly to a level slightly lower than the control. At 42 °C, the *GbHSP16.8* transcription level rose to about 1.58 times higher than the control after 30 min, and then decreased to about 0.47 times that of the control at 6 h. At 8 h, the transcription level was slightly increased; however, the level was still lower than that of the control. It appears that a high temperature (42 °C) inhibits the expression of *GbHSP16.8* (Table S1).

*GbHSP17* was quite sensitive to high temperature. At 36 °C, the transcription level was about 1.58 times that of the control within the first 60 min; after which it dropped to the control level and reached equilibrium. At 40 °C, the transcription level reached a peak after 30 min at 2.94 times that of the control (Figure 4b). The transcription level then decreased slowly after 2 h. At 42 °C, the transcription level rose to 1.52 times that of the control after 30 min and then decreased to 0.77 times that of the control after 8 h (Table S2).

*GbHSP70* was also quite sensitive to high temperatures (Figure 4c). In particular, a high temperature of 42 °C induced a rapid increase in *GbHSP70* transcription. After 30 min, the transcription level rose to 4.21 times that of the control and reached a peak of 4.37 that of the control at 60 min. By 8 h, the transcription level had decreased to a level slightly lower than that of the control. At 40 °C, *GbHSP70* exhibited a slow and constant induction. After 30 min, the transcription level started to increase and reached a peak of 4.26 times that of the control at 4 h. Thereafter, the transcription level decreased slowly and maintained a high transcription level (about 2.96 times that of the control) at 8 h. However, 36 °C had no apparent inductive effect on *GbHSP70*. The transcription level rose slightly at the 30 min time point; however, at 4 h and subsequent time points, the expression level of *GbHSP70* was similar to the control level (Table S3).

Based on these results, *GbHSP16.8* and *GbHSP17* might be induced by low temperature signals. In addition, the two genes might play a key role in the adaptation of *Ginkgo* leaves to low temperatures. *GbHSP70* might also participate in the response to low temperature signals; however, it may have a more important role in the heat stress resistance of *Ginkgo*.

#### 2.1.4. Expression Patterns of the Three *GbHSPs* under Abiotic Stresses

Numerous abiotic stresses are known to trigger alteration in the transcription of HSP genes. Therefore, we determined the expression profiles of the three *HSP* genes under diverse abiotic stress treatments, using QRT-PCR.

**Figure 5:**
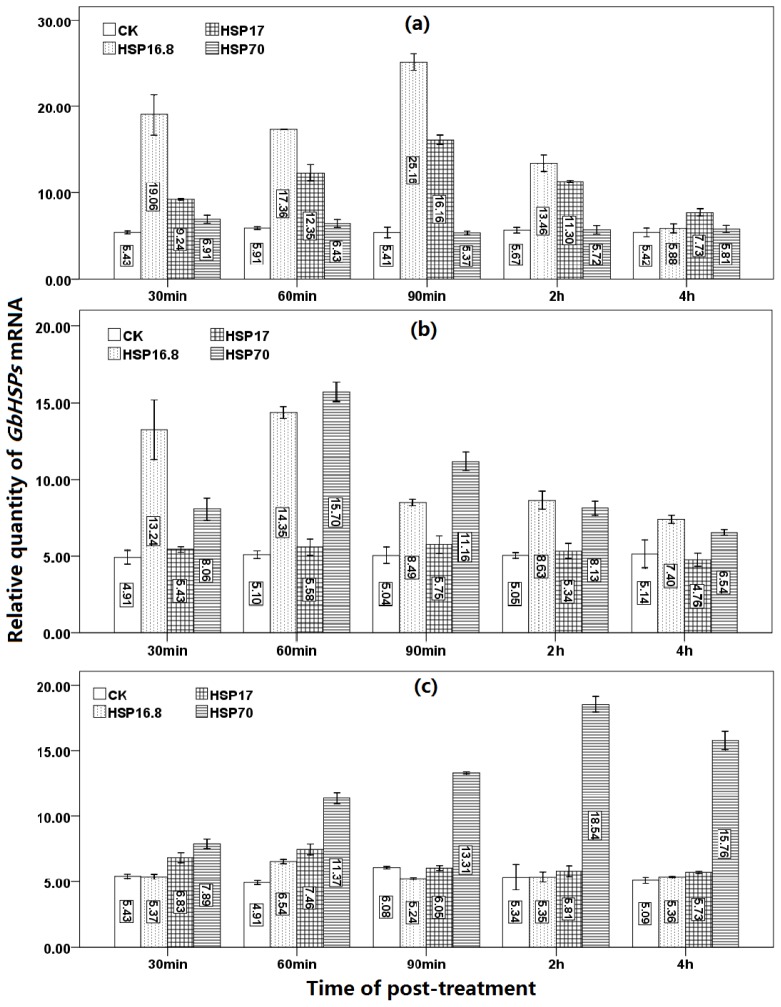
Real-time PCR analysis of the relative expression levels of the *GbHSP* genes in *Ginkgo biloba* under abiotic stress treatment. The *G. biloba GAPDH* gene was used as an internal control. The bars are means of the relative fold change of three biological and two technical replicates obtained by real-time RT-PCR. The standard errors of the biological replicates are shown as error bars. Relative expression levels are shown for (**a**) UV-B treatment, (**b**) wounding treatment and (**c**) ABA treatment.

Irradiation with UV-B upregulated the transcription of the smaller HSPs (Figure 5b). The transcription level of *GbHSP16.8* under ultraviolet treatment varied only slightly after rapidly increasing to 3.51 times that of the control at 30 min. *GbHSP16.8* reached a peak of 4.65 times that of the control level after 90 min, and then slowly decreased. *GbHSP17* was also induced by UV-B, but less efficiently. It reached a peak of 2.98 times higher than the control level after 90 min of treatment. UV-B did not appear to induce the expression of *GbHSP70*. Its expression increased slightly during the early period of treatment and thereafter maintained a similar level to the control (Table S4).

Plants are often injured by environmental factors during their growth and development, and these injuries stimulate plant responses. *GbHSP16.8* and *GbHSP70* were apparently induced by mechanical injuries (Figure 5c). The transcription level of *GbHSP16.8* in leaves rose rapidly 2 h after being cut and reached a peak after 60 min of 2.82 times higher than the control level. The expression level then decreased to 1.44 times lower than the control level, and this level was maintained throughout the remaining experimental period. Injury also plays a key role in the induction of *GbHSP70*. The transcription level rose to 1.64 times higher than the control at 30 min and reached a peak of 3.08 times that of the control after 60 min, after which the expression level slowly decreased. Injury treatment had no apparent role on the induction of *GbHSP17*; the expression level at several time points after treatment did not increase significantly (Table S5).

ABA is one of the five major plant hormones that facilitates the maturity and abscission of fruits and suppresses plant growth. The expression level of the three HSPs increased after ABA treatment (Figure 5a), particularly *GbHSP70*. After 1 h, the transcription level of *GbHSP70* slowly increased to 1.3 times higher than the control level. It further increased to 2.18 times higher than the control level after 90 min and reached a peak of 3.47 times that of the control after 2 h. The expression level then decreased slowly to 3.10 times lower than the control level after 4 h (Table S6). Treatment with ABA had no apparent role in the induction of *GbHSP16.8* (Table S6). The transcription level after about 30 min of treatment was not significantly different to the control level. The transcription level increased slightly after 1 h to 1.33 times higher than the control level, after which it decreased to the control level. The expression profile of *GbHSP17* was similar to that of *GbHSP16.8*.

### 2.2. Discussion

Like other long-lived woody species native to subtropical zone regions, *G. biloba* exhibits a remarkable abiotic stress tolerance. In this study, we cloned three *HSP* genes, particularly *GbHSP16.8*, *GbHSP17*, and *GbHSP70*, from *G. biloba* using SSH cDNA library of *Ginkgo* leaves treated with cold stress and the full sequence was isolated by 5′ and 3′-RACE according to the user manual.

Small heat shock proteins are chaperones that play an important role in stress tolerance [31]. According to their Aa sequence, all cytoplasmic sHSPs in plants described so far belong to two different classes: class I and class II [32]. *GbHSP17* and *GbHSP16.8* belong to cytosolic class I and class II sHSPs and this was validated by the results of alignment and phylogenetic analysis with sHSP sequences of other plants. sHSPs share an evolutionarily conserved sequence of 80–100 amino acids, located in the *C*-terminal region, and called α-crystallin domain or heat shock domain contributing to subunits interactions [33]. The small heat shock domain can be further subdivided into two regions, consensus region I and II separated by a hydrophilic region of variable length. The type of sHSP related with α-crystalline is extremely abundant in higher plants. Though the middle and *N*-terminal amino acids of *GbHSP17* and GbHSP16.8 were very different from others, this difference may partially explain why the two small HSPs could increase resistance to a broader array of stressors than other sHSPs that have been reported.

*Ginkgo HSP16.8 and HSP17* show more homology in the ACD domain, which has two conserved regions. This domain within HSPs is very highly conserved and, as a molecule chaperone, can help to stabilize unfolding proteins because of its propensity to associate with denaturing proteins [34,35]. The conservative sequence (Pro-X-Gly-Val-Leu) is the typical structural feature of most *HSPs*. The structural domain of *GbHSP70* is mainly divided into two parts: the 40 kDa region at the *N*-terminus acts as ATPase and the 30 kDa region at the *C*-terminus functions in the formation of non-foldable polypeptide into protein conformation. Based on HSP16.5 [36] crystal structure of *Methanococcus jannaschii*, we infer that the dimer of HSP is formed by the folding of a large amount of beta sub-units, which are basic structures formed by oligomers. After heat shock, HSP17 (I) and HSP17 (II) will form a particle complex with high molecular weight and a size of 40 nm. These compounds can store housekeeping genes and some degraded protein, e.g., luciferase [37]. HSP70 has a calmodulin binding domain, which contains the important conditioning signal during a living organism’s adaptation to stress [38].

#### 2.2.1. *GbHSPs* Are Expressed under Normal Conditions with Tissue Preference

Most studies have shown that HSPs are needed for basic plant growth and development. However, some previous studies revealed that *HSPs* may only be expressed in a specific tissue under normal growth conditions, partially because of the lesser sensitivity of the Northern blot for very low expression [39]. In the present study, transcripts of the three *GbHSPs* genes were observed in every tissue under general high temperature, although they were only slightly detected in some tissues. The three *GbHSP* genes exhibited diverse expression in different organs, although the expression could be detected in almost all organs we studied. Tissue-specific expression patterns differed from gene to gene even though they belong to the same family as observed in the current study.

In *Arabidopsis*, *HSP70b* transcripts were not detected in any organ; transcripts of *HSP70* were abundant in roots but hardly detectable in other organs; and expression of *HSP70-1* and *HSP70-2* were more abundant in leaves than other organs [40]. Different *HSP* members of the same family can have diverse preferred tissue-specific expression patterns. In rice, *OsHSP80.2* transcripts were detected more abundantly in roots, suggesting a specific role of *OsHSP80.2* in root growth or function. The other two *OsHSP90* genes and *OsHSP70* genes were highly expressed in leaves and sheaths than the other organs [5]. The expression patterns of the three *HSPs* genes in *Ginkgo* were also different. *GbHSP70* and *HSP16.8* were expressed at higher levels in the leaves, which may contribute to maintaining normal leaf functions including respiration and photosynthesis [5]. The other gene was mainly expressed in gynoecia and stamens. *GbHSP70* and *GbHSP16.8* were also expressed at higher levels in stamens, which indicated that they played certain roles in *G. biloba* stamen development.

#### 2.2.2. *GbHSPs* Expression Is Generally Enhanced under Cold or Heat Stress

Expression of *HSP* genes has been shown to be enhanced by elevated temperatures in many plant species [5,40,41]. In *Arabidopsis*, the expression of some *HSP70s* was elevated 2- to 20-fold by 30 min heat stress at 40 °C [40]. In rice, transcripts of some *sHSP-Cl* genes were detected as early as 5 min after exposure to 41 °C treatment. The transcripts of all nine *sHSP-Cl* genes were detected within 15 min [42]. In our present study, transcripts of all three *GbHSPs* genes were increased under heat shock treatment, although they exhibited different response patterns. The transcripts of *GbHSP70* were increased rapidly and kept at constantly high levels during the 40 °C and 42 °C heat stress. The expression of *GbHSP17* was increased 30 min after the 40 °C heat treatment but was reduced after 1 h. The expression of *GbHSP16.8* was increased 30 to 60 min later but was reduced after 4 h. Based on these variations, the different *HSPs* are regulated in different patterns or by different signals. In addition, they may have different assigned functions in response to heat stress. The accumulation of *HSPs* was believed to play a major role in the heat stress response and in acquired thermotolerance in plants [2,43]. The protective effects of HSPs/chaperones can be attributed to the network of the chaperone machinery, in which different HSPs/chaperones acted cooperatively [2].

Although heat shock and cold shock trigger different adaptive responses and induce the production of unique stress proteins, several studies proved that low temperatures could induce *HSPs* involved in the resistance of cells against extreme temperatures [44]. Some *HSP* genes were observed to be induced by low temperature in spinach [45], *Arabidopsis* [40], Sweet chestnut [46], and *Brassica napus* [47]. The study of Soto [48] showed that treatment with low temperature could induce the expression of *CsHSP17.5* in Class I gene in the chestnut cytoplasm, and it could be expressed in both roots and stalks. The expression of *HSP* genes under cold temperature conditions further proved the important role of *HSP* in boosting plant resistance to cold injury [47], Excessive expression of *sHSPs* in the chloroplasts of tomato showed that the symptoms of cold injury in tomato were lesser than the tomato with unexpressed *sHSPs*. The resistance of tomato against cold temperature has improved [49]. However, the specific mechanism of HSPs in inducing resistance of plants to low temperature remains unclear. Guo [22,50] found that the cytoplasmic *sHSP18* gene could be expressed in sweet pepper leaves by subjecting the plant to low temperature, but chloroplast *sHSP26* gene could not be induced by cold stress; Zhu [51] found that *CaHsp24* could be induced by heat stress and also weakly expressed by cold stress. In our study, we found that *GbHSP16.8* and *GbHSP17* could be induced by cold stress, but the *HSP70* gene could not be induced by cold stress. One of the possible reasons may be that only some members of the *sHSP* family respond to low temperature and this does not include in *HSP70*. HSP or other compounds containing HSPs of different molecular weights may be involved in transporting the polypeptide synthesized under cold-induced stress to the plasma membrane, nucleus, and other organelles. Moreover, physiological changes induced by low temperature could denature some functional proteins like the proteins related with membrane fluidity. HSP could induce refolding and restoring the functions of a denatured protein caused by low temperatures [32]. Morimoto [52] proposed the regulating pattern in the expression of *HSP* gene (autoregulation) involving three key steps: *HSF*’s activity is altered by changing internal and external elements; activated *HSF* identifies and binds with *HSE* in the promoter region of the *HSP* gene; and the transcriptionally active region of the *HSP* gene opens and facilitates the transcription.

#### 2.2.3. *GbHSPs* Can Also Be Induced by Stresses Other than Temperature Stress

The three *HSPs* genes isolated from low temperature seedling could be induced by other abiotic stress. Stress induced by UV-B will cause easy accumulation of free radicals and other oxygen derivatives inside the plants. Reactive oxygen-induced stress is an important type of plant stress. In rice seedlings, resistance to UV-B irradiation in rice improved greatly after heat stress treatment. Moreover, rice seedlings with genetically modified *HSP17.7* could improve its resistance to UV-B irradiation, which was related with the transcription level of *HSP17.7* [13]. High temperature and UV-B stress readily cause the formation and accumulation of free oxygen species inside the plants, and further result in oxidation of liposomes, proteins, and other large molecules thereby damaging the plants [53,54]. The expression of *sHSPs* in cytoplasm and mitochondria as induced by active oxygen has been reported [43,55]. When living organisms were subjected to salt tolerance test, sHSPs in the mitochondria could protect the electron transport complex I and prevent its damage by free oxygen. Its role was similar to glutathione, APX, SOD, CAT, and other enzymes [56].

The induction of HSPs by mechanical injury in plants has also been studied, although it has not been fully elucidated. In rice, Oshsp18.0-CII was induced by mechanical injury and SA to a much lower level compared with heat shock. However, mechanical injury and SA did not induce OsHSP 18.0-CII protein accumulation. Mechanical injury could induce the synthesis of enzymes related with the metabolism of phenolic compounds and the production of abundant HSPs in plants. Meanwhile, HSPs could suppress the synthesis of enzymes for phenolic metabolism and prevent the local browning of tissues [57]. In plant roots, sHSPs may contribute to managing periapical lesions, including their influence on the migration of epithelial cell rests and their role in increasing the resistance against necrotic and apoptotic cell death [58]. In *Ginkgo*, damage could induce the synthesis of high amounts of HSP70 and HSP17, indicating that HSPs could be induced solely by mechanical injury without heat stress.

ABA could regulate the adaptation of many plants to environmental stress. The function of ABA in regulating the expression of *HSP* genes in many plants has been widely studied [5,59,60]. In response to drought and temperature stress, ABA levels in plants changed remarkably [61]. When ABA receptor-deficient mutants of maize were subjected to drought, high temperature or both, ABA could improve the endurance of plants by regulating the level of HSP70 synthesis [60]. The transcription level of *HSP70* rose remarkably in response to extreme heat stress. In this study, *GbHSP70* was up-regulated by ABA. The expression of the other two *sHSP* was not affected by ABA, which implied that ABA may improve plant tolerance to extreme heat stress by increasing *HSP70* expression. In rice exposed to heat stress, the promoter structure of all nine *OsHSP* genes has a ABRELATERD1 *cis*-acting element. The adaptation of plants to heat stress was possibly related with the regulation of ABA level [62]. These observations may indicate that both ABA-dependent and independent stress signal transduction pathways were involved in HSP70, HSP16.8 and HSP17 expression regulation. When *Ginkgo* was under extreme high temperature conditions, the transcription levels of HSP70s were related with the regulation of endogenous ABA. HSP16.8 and HSP17 transcription in *Ginkgo* could be induced by high temperatures (36 °C and 40 °C); however, ABA cannot induce the same effect, which implied that *HSP16.8* and *HSP17* genes involved in response to high temperature has no direct relationship with the mechanism involved in regulating ABA.

## 3. Experimental Section

### 3.1. Plant Materials and Treatments

Two-year old grafted *G. biloba* seedlings, growing in a greenhouse in Huanggang (E, 114°54′–116°8′, N, 29°45′–31°35′, Hubei province, central of China), were sampled as cDNA library construction materials. For tissue expression analysis, diverse tissues, including young leaves, mature leaves, ovules, stamens, albumen, gynoecia, stems and roots were collected for RNA extraction as described by Xu [63]. Tissues were immediately frozen in liquid nitrogen and kept at −80 °C prior to total RNA extraction.

Two-year old cuttings from the same genotypic strain of *G. biloba* were subjected to treatments with UV-B, heat-shock, ABA (abscisic acid) and wounding treatment. For UV-B treatment, seedlings were exposed to 1500 μJ/m^2^ UV-B irradiation in a closed chamber, and the control cuttings were placed in a dark closed chamber. The ABA (10 mM) was dissolved in 0.01% Tween 20 and sprayed onto young leaves. The control leaves were sprayed with an equivalent volume of 0.01% (v/v) Tween 20. The edges of the *Ginkgo* leaves were cut by about 0.6 cm with scissors for wounding treatment, the intact leaves of *Ginkgo* were as control. For cold and heat shock treatments, seedlings were exposed to 4 °C and 36 °C, 40 °C, 42 °C. Leaves were collected after exposure for 30 min, 60 min, 90 min, 2 h, 4 h, 6 h, 8 h.

### 3.2. Subtractive Hybridization

Total RNA was isolated from *Ginkgo* seedling leaves of cold treated (4 °C, 1 h, designed as S1) and normal growing conditions (25 °C, designed as S2) using Xu’s method [63]. mRNA was isolated from total RNA with an mRNA Isolation kit (Tiandz, Beijing, China) according to the manufacturer’s instructions. cDNA synthesis, digestion with Rsa I, hybridization, and PCR amplification were carried out using the PCR-Select cDNA subtraction Kit (Clontech, Mountain View, CA, USA) according to the manufacturer’s instructions. Forward subtraction was performed using S2 cDNA as a tester and S1 cDNA as a driver. Reverse subtraction was performed using S1 cDNA as a tester and S2 as a driver. PCR products were ligated into pMD18-T vectors (TaKaRa, Dalian, China) to obtain forward and reverse subtraction libraries. About 2300 colonies each were obtained using a portion of PCR products by SSH in both directions and these ESTs fragments were sequenced by Sangon (Sangon Biotech, Shanghai, China).

### 3.3. Molecular Cloning of the GbHSPs cDNA

Through EST analysis, a 274 bp fragment of *GbHSPs16.8*, 363 bp fragment of *GbHSPs17* and 522 bp fragment of *GbHSP70* were obtained from the heat-treated cDNA library. Based on the sequence, the specific primer pairs (H70R5, H17R5, H16R5 and H70R3, H17R3, H16R3) and the nested primer pairs (H70N5, H17N5, H16N5 and H70N3, H17N3, H16N3) were designed to amplify the 5′ and 3′ end of *GbHSPs* using the SMART™ RACE cDNA Amplification Kit (Clontech, Mountain View, CA, USA). Table 1 lists the primer sequence for each gene. The PCR products were purified and cloned into the pMD18-T vector for sequencing. After comparing and aligning the sequence of 5′RACE, 3′RACE, and the internal fragment, the full-length cDNA sequence of *GbHSPs* were obtained. The full-length cDNA of *GbHSP16.8*, *GbHSP17* and *GbHSP70* were obtained when the 5′ and 3′ fragments were assembled by Vector NTI 10.0 software.

### 3.4. Relative Quantification by QRT-PCR

The transcription levels of GbHSPs were determined in different *G. biloba* tissues, as well as in young seedling leaf samples collected at different time points after stress and hormone treatments. QRT-PCR was carried out using an ABI PRISM 7500 Sequence Detection System (Applied Biosystems**,** Foster City**,** CA, USA) with SYBR Green PCR Master Mix (Applied Biosystems, Foster City**,** CA, USA) according to the manufacturer’s protocol. The *G. biloba* glyceraldehydes-3-phosphate dehydrogenase gene (GbGAPDH, L26924) [64] was used as the reference gene as described by Xu [63].

The gene-specific primers (H70T1, H70T2, H17T1, H17T2, H16T1, H16T2) and reference primers (GAPU, GAPD) for QRT-PCR are listed in Table 1. The QRT-PCR conditions were: 10 min at 95 °C, and 40 cycles (95 °C for 15 s, 60 °C for 1 min). Before performing QRT-PCR, primer efficiency was evaluated using both *GbHSP70* and *GbGAPDH* at 100 nM, 150 nM, 200 nM, 250 nM and 300 nM combinations. A 150 nM concentration was chosen as the most suitable combination for both genes. For each plant sample, aliquots of 150 ng total RNA was analyzed for each gene and the four genes (*GbHSP16.8*, *GbHSP17*, *GbHSP70* and *GbGAPDH*) were always analyzed simultaneously. Each sample was amplified 3 times and all reactions were performed on an ABI PRISM 7500 Sequence Detection System. With a housekeeping gene *GbGAPDH*, the relative amount of the three *GbHSP* transcriptsw is presented as 2(-ddCt) according to the CT method (dCt = Ctsample-Ctcontrol) described in the QRT-PCR Application Guide (Applied Biosystems). When comparing the expression of *GbHSPs* in different tissues, the relative expression of *GbHSPs* was achieved by calibrating its transcription level to that of the reference gene, *GbGAPDH*.

### 3.5. Statistics

Similarity search of the three GbHSP proteins were performed with the Blastx or Blastp program [65]. Multiple sequence alignment of the deduced GbHSPs with other congeneric HSPs was conducted using the clustlx program. The phylogenetic tree was constructed by a neighbor-joining (NJ) method and measured by bootstrap analysis with 1000 replicates. Phylogenetic tree analysis of GbHSPs and known HSPs from other plant species retrieved from GenBank were aligned with Mega 4.0 [66]. Vector NTI Suite 10 was used for sequence alignment and analysis. SPSS 17 was used for statistical analysis and graphing.

## 4. Conclusions

In summary, the analysis of the tissue and environment stress expression profiles of the three *HSP* genes has improved the functional dissection of *Ginkgo HSP* genes. It is possible that appropriate low-temperature treatment will improve the adaptation of *Ginkgo* to other abiotic stresses. Elucidation of the precise role of each *GbHSP* gene, however, requires other experimental approaches including overexpression or RNAi strategies.

## Figures and Tables

**Table 1:** Primers used in the present study.

Primer	Sequence (5′–3′)	Description
H70R5	TGTTCCGCATGTTGTAGGCATAGTT	Reverse primer for 5′RACE, outer
H70R3	CTATTCCCACAAAGAAAGAGCAGGTT	Forward primer for 3′RACE, outer
H17R5	GGTTTAGGTTCAGGTTGTTTAGGCAC	Reverse primer for 5′RACE, outer
H17R3	CCAGGTTTGAAGAAAGAGGAGGTTA	Forward primer for 3′RACE, outer
H16R5	TGCCGACTCTTCGCTCCATTCTTAT	Reverse primer for 5′RACE, outer
H16R3	TAAGAATGGAGCGAAGAGTCGGCAAAT	Forward primer for 3′RACE, outer
H70N5	CTTTGTGGGAATAGTAGTGTTT	Reverse primer for 5′RACE, nested
H70N3	ATGGCATCCTTAATGTCTCA	Forward primer for 3′RACE, nested
H17N5	CGATGCCATTTGTCATTCTT	Reverse primer for 5′RACE, nested
H17N3	CCTGAGAATGCCAAGGTAGA	Forward primer for 3′RACE, nested
H16N5	GCTTTCTCATCTCGCTTTCG	Reverse primer for 5′RACE, nested
H16N3	CGTTACGGTTCCCAAGATTC	Forward primer for 3′RACE, nested
H70T1	ACCAATGACAAGGGTAGG	Primer for QRT-PCR, forward
H70T2	TGTAGGCATAGTTCTCCAAT	Primer for QRT-PCR, reverse
H17T1	CTCACATCTTCAAGGCTGATC	Primer for QRT-PCR, forward
H17T2	CTTCTTCTTTGCTGCGTTCT	Primer for QRT-PCR, reverse
H16T1	GAGCGAAGAGTCGGCAAATT	Primer for QRT-PCR, forward
H16T2	TAGCACGCCATCGTAACACG	Primer for QRT-PCR, reverse
GAPU	TGTCACGGTTTTCGGTTGTAG	Control Primer for QRT-PCR, forward
GAPD	ACCTTTTTGGCACCTCCCTTA	Control Primer for QRT-PCR, reverse
